# Decrease in bone mineral density during three months after diagnosis of early rheumatoid arthritis measured by digital X-ray radiogrammetry predicts radiographic joint damage after one year

**DOI:** 10.1186/s13075-017-1403-0

**Published:** 2017-09-02

**Authors:** Michael Ziegelasch, Kristina Forslind, Thomas Skogh, Katrine Riklund, Alf Kastbom, Ewa Berglin

**Affiliations:** 1 Department of Rheumatology, Department of Clinical and Experimental Medicine, Linköping University, Linköping, Sweden; 2 Section of Rheumatology, Department of Medicine, Helsingborg Hospital, Helsingborg, Sweden; 3 Department of Clinical Sciences, Section of Rheumatology, Lund University, Helsingborg, Sweden; 4 Department of Diagnostic radiology, Umeå university Hospital, Umeå, Sweden; 5 Department of Public Health and Clinical Medicine/Rheumatology, Umeå university Hospital, Umeå, Sweden

**Keywords:** Digital X-ray radiogrammetry, Bone mineral density, Disease progression, Early rheumatoid arthritis

## Abstract

**Background:**

Periarticular osteopenia is an early sign of incipient joint injury in rheumatoid arthritis (RA), but cannot be accurately quantified using conventional radiography. Digital X-ray radiogrammetry (DXR) is a computerized technique to estimate bone mineral density (BMD) from hand radiographs. The aim of this study was to evaluate whether decrease in BMD of the hands (BMD loss), as determined by DXR 3 months after diagnosis, predicts radiographic joint damage after 1 and 2 years in patients with early RA.

**Methods:**

Patients (n = 176) with early RA (<12 months after onset of symptoms) from three different Swedish rheumatology centers were consecutively included in the study, and 167 of these patients were included in the analysis. Medication was given in accordance with Swedish guidelines, and the patients were followed for 2 years. Rheumatoid factor and antibodies to cyclic citrullinated peptides (anti-CCP) were measured at baseline, and 28-joint Disease Activity Score (DAS28) was assessed at each visit. Radiographs of the hands and feet were obtained at baseline, 3 months (hands only) and 1 and 2 years. Baseline and 1-year and 2-year radiographs were evaluated by the Larsen score. Radiographic progression was defined as a difference in Larsen score above the smallest detectable change. DXR-BMD was measured at baseline and after 3 months. BMD loss was defined as moderate when the decrease in BMD was between 0.25 and 2.5 mg/cm^2^/month and as severe when the decrease was greater than 2.5 mg/cm^2^/month. Multivariate regression was applied to test the association between DXR-BMD loss and radiographic damage, including adjustments for possible confounders.

**Results:**

DXR-BMD loss during the initial 3 months occurred in 59% of the patients (44% moderate, 15% severe): 32 patients (19%) had radiographic progression at 1 year and 45 (35%) at 2 years. In multiple regression analyses, the magnitude of DXR-BMD loss was significantly associated with increase in Larsen score between baseline and 1 year (*p* = 0.033, adjusted *R*-squared = 0.069).

**Conclusion:**

DXR-BMD loss during the initial 3 months independently predicted radiographic joint damage at 1 year in patients with early RA. Thus, DXR-BMD may be a useful tool to detect ongoing joint damage and thereby to improve individualization of therapy in early RA.

## Background

Early stages of rheumatoid arthritis (RA) are characterized by gradually developing joint swelling, stiffness and pain, and the patients often have a history of several months of symptoms when first presenting to the rheumatologist. Periarticular bone loss may already be present at this stage, representing an early radiologic manifestation visible on plain radiographs [1, 2]. The disease course in RA shows considerable inter-individual variation, ranging from mild and self-limiting to severe erosive disease, sometimes with extra-articular manifestations. Early treatment with disease-modifying anti-rheumatic drugs (DMARDs) is known to improve disease outcome [3–5], and may limit disease-associated bone loss [6]. However, further improved individual prediction of the disease course and outcome remains an important issue in order to optimize anti-rheumatic therapy.

Digital X-ray radiogrammetry (DXR) is a technique that uses computerized analyses of standard hand radiographs to estimate peripheral bone mineral density (BMD) of the three middle metacarpal bones (DXR-BMD) [7, 8]. DXR-BMD loss has repeatedly been shown to predict radiographic joint progression in early RA [9–14]. However, the majority of previous DXR-BMD studies have been based on 12-month change, and by that time, conventional X-ray assessments of joint damage are at least as informative about disease progression [9, 11–14]. A Dutch study addressing DXR-BMD change after 4 months, reported an independent association between DXR-BMD loss and subsequent radiographic damage [10]. This study was part of a clinical trial with selected patients, and the treatment regimens were slightly different from standard care in Sweden. Therefore, we wished to investigate whether 3-month change in DXR-BMD predicts radiographic joint damage after 1 and 2 years in “real-world” patients with recent-onset RA.

## Methods

### Patients

Patients (n = 176) with early RA (64% women, symptom duration < 12 months), fulfilling the inclusion criteria (see subsequent text) and giving their informed consent, were consecutively included from three Swedish regions (one in Northern and two in Southern Sweden) in 2008–2014 and were followed for 2 years. All patients fulfilled the 2010 American College of Rheumatology (ACR)/European League Against Rheumatism (EULAR) [15] and/or the 1987 ACR [16] classification criteria. Pharmacotherapy was prescribed as found appropriate by the treating rheumatologist, according to Swedish guidelines. Baseline characteristics are detailed in Table 1.

**Table 1:** Baseline characteristics by radiographic progression at 1 year and bone mineral density loss after 3 months

	Total	Radiographic progression			BMD loss		
		No	Yes	*p* value	No	Yes	*p* value
Age	58 (14.5)	57.9 (14.4)	60.3 (14.7)	0.41	55.7 (15.5)	60.3 (13.4)	*0.042*
Women, *n* (%)	107 (64)	84/135 (62.2)	23/32 (71.9)	0.41	38/69 (55.1)	69/98 (70.4)	0.05
Symptom duration (months)	6 (3.7)	5.5 (3.3)	6.4 (5.0)	0.35	6 (3.2)	5.5 (3.9)	0.31
Anti-CCP2 positive, *n* (%)	107 (64)	88/135 (65.2)	19/32 (59.4)	0.54	42/69 (60.8)	65/98 (66.3)	0.51
RF positive, *n* (%)	103 (63)	83/131 (63.4)	20/32 (62.5)	1.0	40/68 (58.8)	63/95 (66.3)	0.41
ESR (mm/h)	29 (21.4)	27.3 (21.1)	35.4 (21.8)	0.055	25.4 (18.2)	31.3 (23.2)	0.066
CRP (mg/ml)	22 (24.4)	21.6 (25.0)	24.7 (22.2)	0.51	18.2 (23)	25 (25.1)	0.074
DAS 28	4.86 (1.30)	4.79 (1.3)	5.12 (1.4)	0.21	4.57 (1.27)	5.06 (1.29)	*0.023*
HAQ	0.94 (0.60)	0.91 (0.59)	1.08 (0.59)	0.15	0.86 (0.55)	1 (0.62)	0.16
Larsen total	4.1 (4.9)	3.57 (4.63)	6.25 (5.46)	*0.005*	3.16 ((4.23)	4.73 (5.24)	*0.034*
DXR-BMD	579.5 (86.1)	584.7 (84.2)	558.0 (91.7)	0.115	600.6 (84.2)	564.8 (84.7)	*0.008*
DXR-BMD loss	0.68 (1.81)	−0.52 (1.65)	−1.37 (2.31)	0.058	0.83 (1.05)	−1.74 (1.45)	*< 0.001*

Values are mean (SD) unless otherwise stated

*Abbreviations*: *CCP* cyclic citrullinated peptides, *RF* rheumatoid factor, *ESR* erythrocyte sedimentation rate, *CRP* C-reactive protein, *DAS28* Disease Activity Score, *HAQ* Health Assessment Questionnaire, *DXR-BMD* bone mineral density measured by digital X-ray radiogrammetry

*p* values in italics are statistically significant

At baseline, 83% of patients were prescribed oral prednisolone, 49% received osteoporosis prophylaxis with low-dose calcium phosphate and vitamin D, and 6% with bisphosphonates. 91% received conventional synthetic DMARDs (csDMARD) (88% methotrexate, 2.4% other csDMARDs and 0.6% combination therapy). One patient (0.6%) was started on a tumor necrosis factor (TNF) inhibitor at baseline. During the follow-up period 14.4% received biologic therapy (bDMARDs) as displayed in Table 2.

**Table 2:** Treatment characteristics

Treatment	Number (percentage) of patients
csDMARDs/biologics started at baseline, *n* (%)	152 (91.0)
- MTX, *n* (%)	147 (88.0)
- Other csDMARDs, *n* (%)	4 (2.4)
- csDMARD triple therapy, *n* (%)	1 (0.6)
Anti-TNF at baseline, *n* (%)	1 (0.6)
Anti-TNF ever, *n* (%)	18 (10.8)
Other bDMARDs	6 (3.6)
Oral glucocorticoids, *n* (%)	138 (82.6)
Calcium supplements, *n* (%)^a^	79 (49.1)
Bisphosphonates, *n* (%)^b^	7 (6.0)

*Abbreviations*: *MTX* methotrexte, *cDMARDs* conventional disease modifying anti-rheumatic drugs, *TNF* tumor necrosis factor, *bDMARDs* biologic disease modifying anti-rheumatic drugs

^a^Data available for 161 patients

^b^Data available for 124 patients

### Radiographic assessment and digital X-ray radiogrammetry (DXR)

Radiographs (posterior-anterior projection) of the hands, wrists and forefeet were performed at baseline, 3 months (hands only) and 1 and 2 years. The baseline and 1-year and 2-year radiographs were read in chronological order and evaluated according to the Larsen score [17] by one investigator at each center (MZ, KF and EB). The scoring system included 32 areas; metacarpal-phalangeal joints II − V, all proximal interphalangeal joints, the wrists divided into four areas and the metatarsophalangeal joints II–V. Each joint and joint area was graded 0–5. The maximum total score was 160. The smallest detectable change (SDC) was calculated for the three readers individually (EB, 2; KF, 1; MZ, 3) according to the method of Bruynesteyn [18]. Radiographic progression was defined as a difference in Larsen score above the SDC of the corresponding reader. The intra-rater and inter-rater reliability of the readers was assessed by calculating the intraclass correlation coefficient (ICC). The ICC was 0.903.

BMD was estimated on hand radiographs of the second, third and fourth metacarpal bones using DXR (the online Pronosco X-posure System, SECTRA, Linköping, Sweden), a computerized version of the traditional technique of radiogrammetry measuring cortical bone thickness [8, 19]. DXR-BMD was assessed at inclusion and after 3 months. The mean value of both hands was used in all analyses. DXR-BMD values are given in mg/cm^2^. DXR-BMD loss was categorized either as a moderate decrease in DXR-BMD (≥ 0.25 but < 2.5 mg/cm^2^ per month) or a severe decrease (≥ 2.5 mg/cm^2^ per month), as defined by the provider (Sectra) [20]. To ensure consistent image acquisition, the images for each patient were always taken in a frontal position of the hands using the same X-ray machine. The images were sent unprocessed to Sectra for DXR analysis.

### Clinical and laboratory assessments

The erythrocyte sedimentation rate (ESR, mm/1 h) and C-reactive protein (CRP, mg/L) were determined at baseline and after 3, 6, 12 and 24 months. At the same time points, the 28-joint Disease Activity Score (DAS28) was calculated by the patient’s regular physician [21]. Therapy response was determined according to EULAR response criteria [22]. Functional status was evaluated using the Swedish version of the Stanford Health Assessment Questionnaire (HAQ) [23]. Rheumatoid factor (RF) and antibodies to cyclic citrullinated peptides (anti-CCP2) were analyzed in baseline serum samples at the clinical immunology units of the local hospitals.

### Statistics

Statistical calculations were performed using SPSS software (version 23, IBM Corporation, Armonk, USA). Linear regression analyses were used to explore the effect of DXR-BMD alone, and in combination with various clinical and laboratory variables, chosen with respect to clinical assumptions, for associations with change in Larsen score after 1 and 2 years. After testing each variable in a simple regression analysis with change in Larsen score at 1 and 2 years as the dependent variable, multiple regression analyses were performed including variables with *p* < 0.2. Radiographic progression was defined as a difference in Larsen score above the SDC between baseline and 1 and 2 years, respectively. Thus, for example, radiographs assessed by KF with a difference > 1 between the two time points were graded as the actual difference, otherwise as 0. As sensitivity analyses, we also performed linear regression with the same variables, but with absolute changes in Larsen, i.e. not considering the SDC. In addition, logistic regression analysis was performed, in which radiographic progression was defined as change in Larsen score greater than SDC. The Pearson chi^2^ test or Fisher’s exact test were used for categorical variables, and the independent samples *t* test was used for continuous variables. All *p* values are two-sided, and *p* values less than 0.05 were considered statistically significant.

## Results

**Fig. 1:**
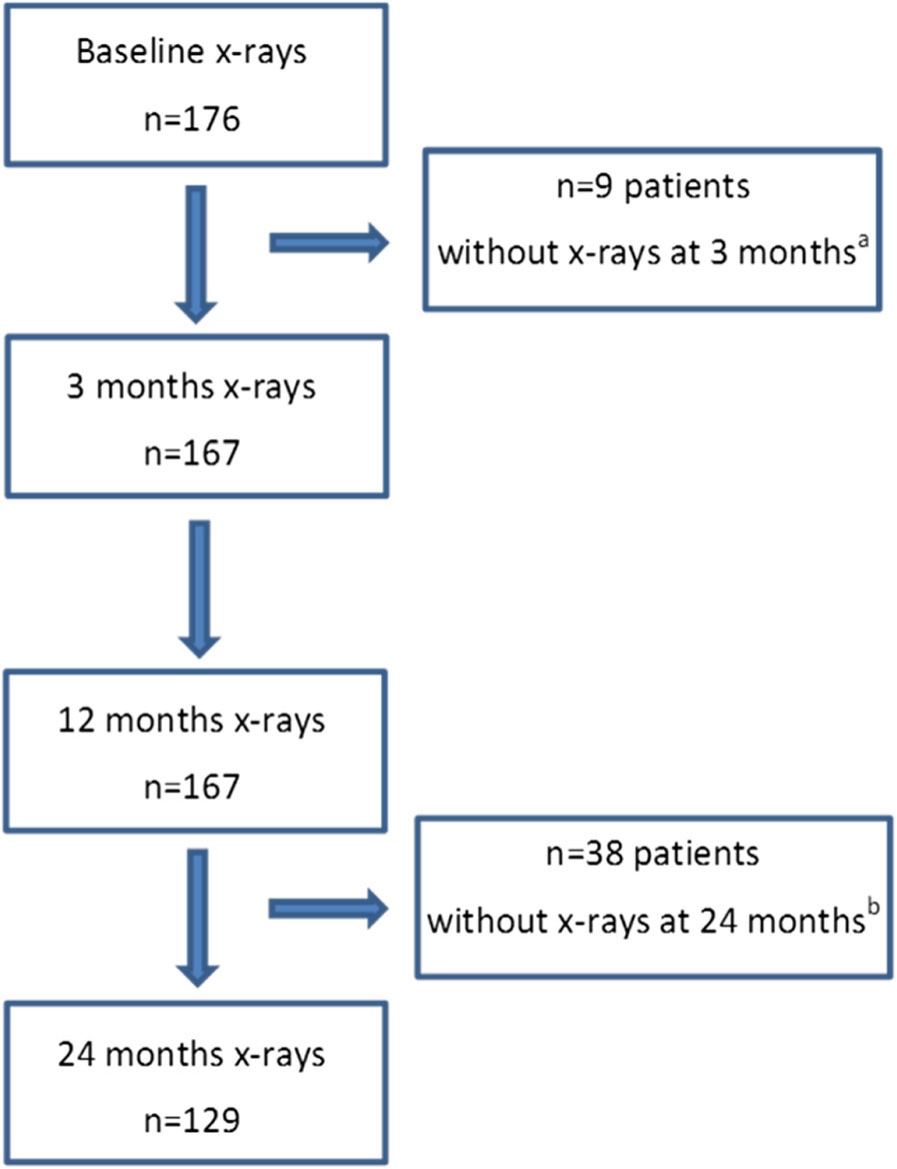
Availability of radiographs at baseline and follow up. There were 176 patients included in the study. ^a^Nine patients were lost because of missing radiographs at 3 months, and thus, the evaluation included 167 patients with radiographs at baseline, 3 months and 1 year. ^b^Of the 167 patients included in the evaluation, 129 also underwent radiography at 2 years

In total, 176 patients without previous DMARD exposure were included in the study. Table 1 shows the patient characteristics and Table 2 the anti-rheumatic therapy including glucocorticoids (GC), and treatments that influence BMD, initiated at baseline. There were no significant differences in these background characteristics between the participating sites (data not shown). Nine patients were lost because of missing radiographs at 3 months. Thus, the evaluation included 167 patients with radiographs at baseline, 3 months and 1 year (Helsingborg, n = 38; Linköping, n = 65; Umeå, n = 64). Of the 167 patients, 129 also underwent radiography at 2 years (Fig. 1). Compared with the patients with 2-year radiographs available, patients without 2-year radiographs had lower mean Larsen score at baseline (2.1 (SD = 3.17) vs 4. 7 (SD = 5.17); *p* < 0.001) and at 1 year (2.6 (SD = 3.34) vs 5.8 (SD = 5.66); *p* < 0.001), and were to a lesser extent RF positive (48.6% vs 67.5%; *p* = 0.037). Other baseline characteristics, as detailed in Tables 1 and 2 did not significantly differ from those in patients with radiographs available at 2 years.

At 3 months, 105 (63%) patients had low disease activity (DAS28 ≤ 3.2) and 80 (48%) had reached EULAR remission (DAS28 ≤ 2.6): 46 (28%) of the patients had moderate and 6 (4%) high disease activity (DAS28 > 5.2). DAS28 values from the 3-month visits were missing for 10 (6%) patients. After 1 year, 108 (65%) of the patients had low disease activity, 36 (22%) patients had moderate and 7 (4%) high disease activity: 88 (53%) of the patients had reached EULAR remission. DAS28 values from the 1-year visit were missing in 16 patients (10%).

The mean age of the male patients (n = 60) at baseline was 61 years (SD = 14.5) and the mean age of the female patients (n = 107) was 57 years (SD = 14.3). Comparing our DXR-BMD values with a Danish reference cohort of healthy individuals [24], 92 patients (55.1%) in our cohort had bone loss in the hand exceeding the age-related bone loss in the hand among the Danish controls.

Of the 167 patients, 32 (19%) had radiographic progression at 1 year and 45 of 129 patients (35%) had radiographic progression at 2 years. The change in DXR-BMD over 3 months showed BMD loss in 98 patients (59%). The DXR-BMD loss was moderate in 73/167 patients (44%) and severe in 25/167 patients (15%). Radiographic joint damage was significantly different across the three categories of DXR-BMD loss at baseline and at 1 year (*p* = 0.039 and *p* = 0.024, respectively) and there was a trend towards statistical significance after 2 years (*p* = 0.056) (Table 3). Categorizing DXR-BMD loss according to the age-related reference material presented by Ornbjerg et al. [24] yielded very similar results (data not shown).

**Table 3:** Mean Larsen scores and 3-month bone mineral density loss in early rheumatoid arthritis

	Mean (SD) Larsen score			*p* value
	No BMD loss	Moderate BMD loss	Severe BMD loss	
Baseline (SD)	3.2 (4,23)	4.3 (4.81)	6.0 (6.28)	0.039
1 year (SD)	4.0 (4.68)	5.3 (5.45)	7.3 (6.38)	0.024
2 years (SD)	5.4 (5.1)	6.9 (5.6)	8.8 (6.04)	0.056

*BMD* bone mineral density

Patients with change in Larsen score greater than the SDC after 1 year had significantly higher Larsen scores (mean) at baseline (3.6 vs 6.2; *p* = 0.005). Compared with patients without DXR-BMD loss, patients with DXR-BMD loss after 3 months were significantly older (60.3 years vs 55.7; *p* = 0.042), had significantly higher baseline DAS28 (5.1 vs 4.6; *p* = 0.023) and significantly higher Larsen scores at baseline (4.7 vs 3.2; *p* = 0.034). Also, the proportion of women was significantly higher (70.4% vs 55.1%; *p* = 0.05) among patients with BMD loss (Table 2). There was no significant difference in the DXR value at baseline in the anti-CCP2-positive compared with the anti-CCP-negative patients (576 vs 587 mg/cm^2^; *p* = 0.426).

Simple regression analyses with change in Larsen score greater than the SDC at 1 year as the dependent variable were performed, including the following covariates: age, sex, oral corticosteroid treatment, DXR-BMD loss/month, baseline DAS28, CRP, ESR, Larsen score, anti-CCP2 status, and RF status. Also, DAS28 > 2.6 at 3 months (yes/no) was included. Covariates with a *p* value < 0.2 in these analyses were included in a multiple regression model (Table 4). This model, adjusting for sex and baseline values of ESR, DAS28, Larsen score and anti-CCP2 status, showed a significant association between 3-month BMD loss and increase in Larsen score above the SDC after 1 year (*p* = 0.033, adjusted *R*-squared = 0.069) (Table 4). No significant association was observed between early bone loss and increase in the Larsen score above the SDC at 2 years (*p* = 0.604). When using the same covariates but with change in Larsen score without considering the SDC as the dependent variable, DXR-BMD loss was significantly associated with the 1-year Larsen score (*p* = 0.048), but not the Larsen score at 2 years (*p* = 0.491). In logistic regression analysis, there was no significant association between DXR-BMD loss and 1-year radiographic progression defined as a change in Larsen score above the SDC (*p* = 0.158). Treatment with bisphosphonates, calcium and vitamin D did not influence the DXR-BMD loss (data not shown).

**Table 4:** Regression analyses with change in Larsen score between baseline and 12 months as dependent variable

	Simple regression			Multiple regression		
Variable	Adjusted *R*-square	β-coeff	*p* value	Adjusted *R*-square	β-coeff	*p* value
DXR decrease (mg/cm^2^/month)^a^	0.049	−0.235	0.002	0.069	−0.181	0.033
DAS28^b^	0.015	0.148	0.070		0.028	0.767
Larsen score^b^	0.017	0.153	0.049		0.068	0.404
Age^b^	0.001	0.082	0.291			
Gender	0.007	0.114	0.143		0.071	0.381
anti-CCP2 status^b^	0.010	−0.125	0.107		−0.128	0.114
RF^b^	−0.001	−0.069	0.385			
Corticosteroid treatment	−0.002	0.042	0.417			
Disease duration (months)^b^	−0.006	0.026	0.739			
CRP (mg/L)^b^	−0.004	0.045	0.566			
ESR (mm/h)^b^	0.024	0.173	0.025		0.133	0.165
DAS28 > 2.6 at 3 months	−0.002	0.065	0.422			

*Abbreviations*: *DXR* digital X-ray radiogrammetry, *DAS28* Disease Activity Score in 28 joints, *CCP* cyclic citrullinated peptides, *RF* rheumatoid factor, *CRP* C-reactive protein, *ESR* erythrocyte sedimentation rate, *BMD* bone mineral density

^a^Decrease in DXR-BMD between baseline and 3 months

^b^Baseline value

When analyzing only patients without erosions at baseline (n = 123), using the same multivariate model (adjusted for the same variables but not for the Larsen score at baseline), 3-month DXR-BMD loss remained associated with radiographic progression after 1 year (*p* = 0.021, *R*-squared = 0.07). Also, when analyzing the 1-year outcome among the 129 patients with 2-year radiographs available, the association between DXR-BMD loss and radiographic progression remained statistically significant (*p* = 0.039, adjusted *R*-squared = 0.08).

## Discussion

To our knowledge, this is the first study addressing the predictive value of 3-month DXR-BMD in patients with recent-onset RA compared with radiography and clinical data. In clinical practice, evaluation of prescribed DMARDs is commonly performed 3 months after initiation. At this occasion, particularly in early disease, additional information on the patient’s radiographic prognosis would be highly valuable in order to optimize therapy decisions. In this study, we found DXR-BMD loss during the first 3 months to independently predict radiographic joint damage at 1 year and the 1-year progression from baseline.

Our results on metacarpal bone loss among patients with early RA are in line with previous reports [9–14]. The shortest interval of DXR-BMD assessments among previous studies was 3 months in the study from Bøyesen et al. [25], addressing 3-month change in DXR-BMD as a predictive factor for erosive progression identified on magnetic resonance imaging (MRI) in patients with early RA. In the 53 patients completing that study there was only a trend towards higher MRI synovitis score and 3-month DXR BMD loss in patients developing MRI erosions, and no significant changes. Wevers-de Boer and coworkers [10] presented 4-month data with similar findings on the 1-year radiographic outcome as in the current study. Thus, early DXR-BMD assessments seem to be of clinical value, in order to optimize early institution of anti-rheumatic pharmacotherapy and thereby diminish the risk of future disability [4, 26–30]. However, we found no predictive value of DXR-BMD loss in relation to the 2-year radiographic outcome. This was somewhat surprising, since existing radiographic damage often predicts radiographic progression. One possible explanation for the disparate 1-year and 2-year findings in our study could be that potent instituted pharmacotherapy attenuated radiographic differences over time. Also, missing data from 2-year radiographs (n = 38) need to be considered, but the association between DXR-BMD loss and radiographic damage at 1 year remained statistically significant, also after excluding the 38 patients without 2-year radiographs. Thus, difference in statistical power appears to be an unlikely explanation for the discordance between 1-year and 2-year data. A previous study by Forslind et al. [9] showed that patients with early RA, who were on prednisolone 7.5 mg per day in addition to conventional DMARDS, had significantly less DXR-BMD loss as compared with patients with RA who were not receiving corticosteroids. This finding was attributed to the anti-inflammatory effect of prednisolone, hampering osteopenia induced by inflammation. Although not primarily designed to address this, our study did not identify a significant impact of oral corticosteroid on BMD loss or radiographic progression. Similarly, treatment with bisphosphonates, calcium and vitamin D did not significantly impact BMD loss.

In our study we did not observe a significant difference in the DXR value at baseline in the anti-CCP2-positive compared with the anti-CCP-negative patients. This fact is contrary to the findings in other studies [31–33] in which BMD loss was significantly more widespread in anti-CCP-positive patients. Different methods for estimating BMD loss (comparative micro computed tomography (micro-CT) analysis and dual-energy X-ray absorptiometry) may influence the differing results. Also, it needs to be pointed out that our study was not primarily designed to address the influence of anti-CCP on BMD loss.

Whenever a patient with RA presents with erosions at the time of diagnosis, an aggressive disease course is assumed, and treatment is chosen accordingly. Thus, information on the radiographic prognosis is even more important in the large category of patients without evidence of erosive disease at diagnosis. Interestingly, we found that 3-month DXR-BMD loss also predicted 1-year radiographic damage in this subgroup of patients. However, the comparative value of DXR-BMD compared with other imaging modalities needs to be assessed.

Limitations of this study are the missing 2-year radiographs in 38 patients (23%), and the fact that the baseline Larsen score and RF status differed between patients with radiographs available at 2 years and those without. Nevertheless, analyzing the 129 patients with all radiographs available up to 2 years did not substantially alter the findings.

## Conclusion

In this real-world study of patients with early RA, we found that DXR-BMD loss during the initial 3 months independently predicted radiologic damage at 1 year. However, DXR-BMD loss predicts only a minor part of the variation in radiographic damage, and an association was not established after 2 years of disease. Future studies should compare the value of DXR-BMD with other imaging modalities.

## Abbreviations

ACR: American College of Rheumatology
bDMARD: Biologic disease modifying anti-rheumatic drug
BMD: Bone mineral density
CCP: Cyclic citrullinated peptides
csDMARD: Conventional synthetic disease modifying anti-rheumatic drug
CRP: C-reactive protein
DAS: Disease Activity Score
DAS28: DAS with 28 joints
DXR: Digital X-ray radiogrammetry
DXR-BMD: Bone mineral density measured by digital X-ray radiogrammetry
ESR: Erythrocyte sedimentation rate
EULAR: European League Against Rheumatism
GC: Glucocorticoid
HAQ: Health Assessment Questionnaire
ICC: Intraclass correlation coefficient
MRI: Magnetic resonance imaging
MTX: Methotrexate
RA: Rheumatoid arthritis
RF: Rheumatoid factor
SDC: Smallest detectable change
TNF: Tumor necrosis factor
